# Bisphenol-A Affects Male Fertility via Fertility-related Proteins in Spermatozoa

**DOI:** 10.1038/srep09169

**Published:** 2015-03-16

**Authors:** Md Saidur Rahman, Woo-Sung Kwon, June-Sub Lee, Sung-Jae Yoon, Buom-Yong Ryu, Myung-Geol Pang

**Affiliations:** 1 Department of Animal Science and Technology, Chung-Ang University, Anseong, Gyeonggi-do 456-756, Republic of Korea

## Abstract

The xenoestrogen bisphenol-A (BPA) is a widespread environmental contaminant that has been studied for its impact on male fertility in several species of animals and humans. Growing evidence suggests that xenoestrogens can bind to receptors on spermatozoa and thus alter sperm function. The objective of the study was to investigate the effects of varying concentrations of BPA (0.0001, 0.01, 1, and 100 μM for 6 h) on sperm function, fertilization, embryonic development, and on selected fertility-related proteins in spermatozoa. Our results showed that high concentrations of BPA inhibited sperm motility and motion kinematics by significantly decreasing ATP levels in spermatozoa. High BPA concentrations also increased the phosphorylation of tyrosine residues on sperm proteins involved in protein kinase A-dependent regulation and induced a precocious acrosome reaction, which resulted in poor fertilization and compromised embryonic development. In addition, BPA induced the down-regulation of β-actin and up-regulated peroxiredoxin-5, glutathione peroxidase 4, glyceraldehyde-3-phosphate dehydrogenase, and succinate dehydrogenase. Our results suggest that high concentrations of BPA alter sperm function, fertilization, and embryonic development via regulation and/or phosphorylation of fertility-related proteins in spermatozoa. We conclude that BPA-induced changes in fertility-related protein levels in spermatozoa may be provided a potential cue of BPA-mediated disease conditions.

Bisphenol-A (BPA) is an endocrine disruptor (ED) that possesses weak estrogenic, anti-androgenic, and anti-thyroid activities, and it is a prominent environmental pollutant due to its use in the manufacture of food packaging, industrial materials, and dental sealant^1^. Owing to its extensive use, toxicity, and environmental persistence, BPA has become a significant public health concern. The major route of BPA exposure for humans and animals is ingestion of contaminated food and water, but exposure can also occur via dermal and respiratory routes^2^.

BPA is associated with the impairment of male reproductive function, as well as diabetes, obesity, cardiovascular disease, thyroid dysfunction, developmental disorders, miscarriages, female reproductive abnormality, and cancer^3^. Most studies on BPA in the context of male reproduction/fertility have been conducted *in vivo*, and these studies have consistently reported its developmental toxicity to spermatozoa via DNA damage^4^, DNA methylation^5^, oxidative stress^6^, and lipid peroxidation^7^, among other mechanisms. While few *in vivo* studies have shown an association between BPA and spermatozoa functional parameters, BPA has been linked to changes in motility^8^, morphology^9^, and intracellular ATP content^6^.

Earlier studies have described the presence of nongenomic estrogen receptors on sperm cell membranes, which interfere with sperm functions upon stimulation with estradiol^10^,^11^,^12^. These results show that spermatozoa may represent a valid model for studying the effects of estrogenic EDs like BPA *in vitro*. This model may allow further exploration of previous *in vivo* findings via investigation of the effects of BPA on the functional maturation process of mammalian spermatozoa, which includes motility, hyperactivation, capacitation, and the acrosome reaction. This functional maturation process is important because it confers the ability to fertilize oocytes^13^. The effect of BPA on consequent fertilization and embryo development should also be investigated.

The recent application of proteomic approaches has led to the discovery of several spermatozoan proteins, some of which have been associated with fertility^14^,^15^. However, the precise functions of spermatozoan proteins in fertility/infertility are not well understood^16^. It is difficult to draw conclusions from the results of studies conducted in models that do not use spermatozoa, because proteins can perform functions in the whole organism that differ from their functions in spermatozoa^17^. Thus, to understand the molecular mechanisms that regulate sperm function under environmental stress, intensive studies on individual protein function are needed. Therefore, the objectives of the present study were as follows: (1) to determine whether BPA exposure alters spermatozoa function and identify the underlying molecular mechanisms and (2) to investigate the function of fertility-related proteins in spermatozoa under BPA exposure *in vitro*. We selected the following fertility-related proteins based on previous results: β-actin^18^, peroxiredoxin-5 (PRDX5)^19^, glutathione peroxidase 4 (GPX4)^20^, glyceraldehyde-3-phosphate dehydrogenase (GAPDH)^21^, and succinate dehydrogenase (SDHB)^22^. Additionally, we evaluated the functional interactions of the selected fertility-related proteins in spermatozoa with regard to the regulation of sperm function, fertilization, and the fate of the embryo.

## Results

### Effect of BPA on sperm motility and motion kinematics

**Figure 1:**
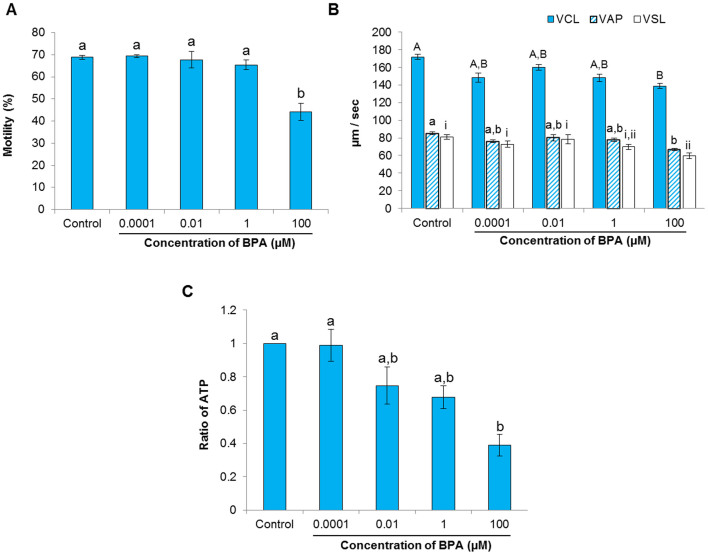
Effect of bisphenol-A (BPA) on motility parameters and intracellular ATP levels of spermatozoa. (A) Differences in spermatozoa motility after incubation with different concentrations of BPA compare to control. (B) Differences in motion kinematics parameters such as curvilinear velocity (VCL, blue bar), average path velocity (VAP, stripped bar), and straight line velocity (VSL, open bar) between the control and BPA-treated samples. (C) Differences in ATP levels between the control and BPA-treated samples. Data are presented as mean ± SEM (4 replicates). Values with different superscript characters (^A,B,a,b,i,ii^) indicate significant difference between the control and treatment groups as determined by one-way ANOVA (*P* < 0.05).

Sperm motility parameters are associated with fertilization and pregnancy rates^23^,^24^,^25^; therefore, the effect of BPA on sperm motility parameters was evaluated. An initial time-dependent experiment (ranging from 0.5 to 8 h) was conducted to detect sperm motility in the control group and the high-concentration (100 μM) treatment group to allow us to choose the optimal incubation time (see Supplemental Material, Fig. S1). Motility parameters were measured following 6 h of incubation with ascending concentrations of BPA (0.0001, 0.01, 1, and 100 μM). The percentage of motile spermatozoa decreased significantly in the presence of 100 μM BPA (*P* < 0.05; Fig. 1A). BPA at 100 μM also significantly reduced curvilinear velocity, average path velocity, and straight-line velocity (*P* < 0.05; Fig. 1B).

### The effect of BPA on intracellular ROS content and ATP levels in spermatozoa

To evaluate the effect of BPA on intracellular *ROS* content and ATP levels in spermatozoa, both were quantitatively detected as described in the Materials and Methods. A significant decrease in ATP production occurred in spermatozoa treated with 100 μM BPA (*P* < 0.05; Fig. 1C). However, a non-significant change in ROS production was observed in spermatozoa treated with BPA in comparison with the control (Supplemental Material, Fig. S2).

### Effect of BPA on sperm capacitation status

**Figure 2:**
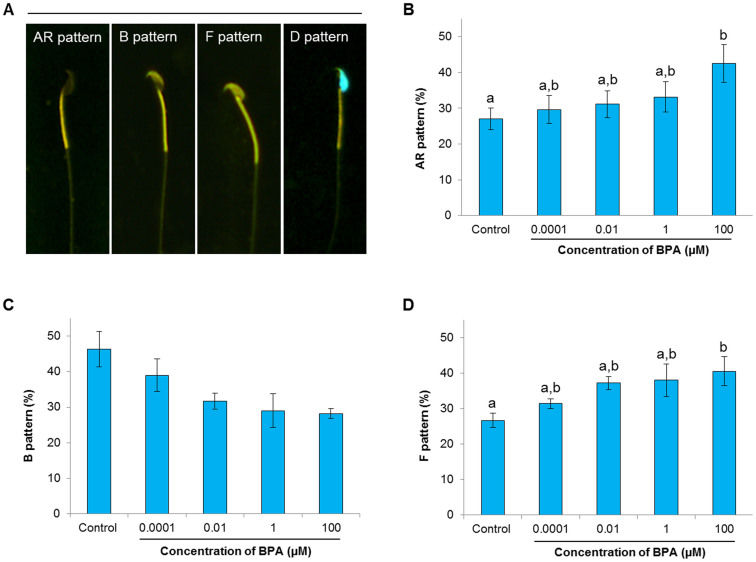
Effect of bisphenol-A (BPA) on the capacitation status of spermatozoa. (A) Different patterns of spermatozoa in combined Hoechst 33258/chlortetracycline fluorescence staining as a result of BPA treatment such as, acrosome-reacted (AR pattern), capacitated (B pattern), non-capacitated (F pattern), and dead (D pattern). (B) AR pattern spermatozoa in the control and BPA-treated samples. (C) B pattern spermatozoa in the control and BPA-treated samples. (D) Differences in the number of F pattern spermatozoa as a result of BPA treatment compare to control. Data are presented as mean ± SEM (3 replicates). Values with different superscript characters (^a,b^) indicate significant difference between the control and treatment groups as determined by one-way ANOVA (*P* < 0.05).

The capacitation status of mammalian spermatozoa is a useful predictor of male fertility^26^,^27^,^28^. In the present study, a dual-staining method was performed to evaluate changes in CTC fluorescence patterns related to sperm capacitation due to *in vitro* exposure to BPA. Increased numbers of acrosome-reacted (AR pattern) spermatozoa were noted after treatment with 100 μM BPA (*P* < 0.05; Fig. 2B). In contrast, the number of capacitated (B pattern) spermatozoa showed a non-significant tendency to decrease as the BPA concentration increased (Fig. 2C). BPA treatment also dose-dependently increased the number of non-capacitated (F pattern) spermatozoa, with marked effects achieved using 100 μM BPA (*P* < 0.05; Fig. 2D).

### BPA-induced protein kinase A (PKA)-dependent phosphorylation of tyrosine residues on proteins in spermatozoa

**Figure 3:**
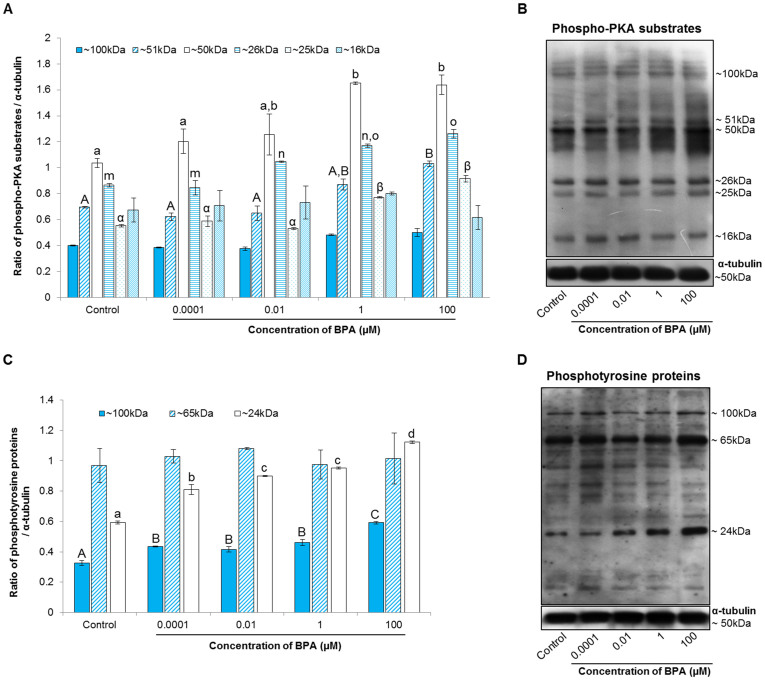
Effect of bisphenol-A (BPA) on protein kinase A (PKA) activity and tyrosine phosphorylation in spermatozoa. (A) Density of phospho-PKA substrates (approximately 100, 51, 50, 26, 25, and 16 kDa) in control and BPA-treated spermatozoa. (B) Phospho-PKA substrates were probed with an anti-phospho-PKA antibody. Lane 1: control; lane 2: 0.0001 μM BPA; lane 3: 0.01 μM BPA; lane 4: 1 μM BPA; and lane 5: 100 μM BPA. (C) Density of phosphotyrosine proteins (approximately 100, 65, and 24 kDa) in the control and BPA-treated samples. (D) Phosphotyrosine proteins were probed with PY 20 antibody. Lane 1: control; lane 2: 0.0001 μM BPA; lane 3: 0.01 μM BPA; lane 4: 1 μM BPA; and lane 5: 100 μM BPA. Data are presented as mean ± SEM (4 replicates). Values with different superscript characters (^A,B,C,a,b,c,d,α,β,m,n,o^) indicate significant difference between the control and treatment groups as determined by one-way ANOVA (*P* < 0.05).

To evaluate the molecular mechanism underlying the BPA-induced acrosome reaction, we measured PKA activity and phosphotyrosine level in spermatozoa by western blotting^25^. Our results showed that the levels of 6 different PKA substrate species (approximately 16, 25, 26, 50, 51, and 100 kDa) increased dose-dependently compared with their levels in the control cells, with particularly noticeable increases in the species weighing approximately 25, 26, 50, and 51 kDa (*P* < 0.05; Fig. 3A, B). Similarly, the tyrosine-phosphorylated species of approximately 24 and 100 kDa increased significantly due to BPA treatment compared with the corresponding levels in the control group (*P* < 0.05; Fig. 3C, D).

### Effect of BPA on fertilization and embryonic development

**Figure 4:**
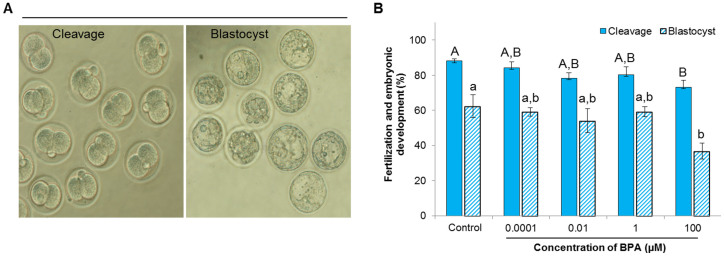
Effect of bisphenol-A (BPA) on fertilization and embryonic development. (A) Representative image of cleavage (2 blastomeres containing polar bodies) and blastocyst (embryonic development at day 5). (B) The rate of fertilization (cleavage) and embryonic development (blastocyst) in control and BPA-treated samples (blue bar: cleavage rate; stripped bar: blastocyst rate). Data are presented as mean ± SEM (3 replicates). Values with different superscript characters (^A,B,a,b^) indicate significant difference between the control and treatment groups as determined by one-way ANOVA (*P* < 0.05).

Next, we used an *in vitro* fertilization system to evaluate the effect of BPA on fertilization and early embryonic development. The rates of cleavage (fertilization, Fig. 4A) and blastocyst (early embryonic development, Fig. 4A) formation were significantly decreased in spermatozoa treated with 100 μM BPA (*P* < 0.05; Fig. 4B).

### Effect of BPA on selected fertility-related proteins in spermatozoa

**Figure 5:**
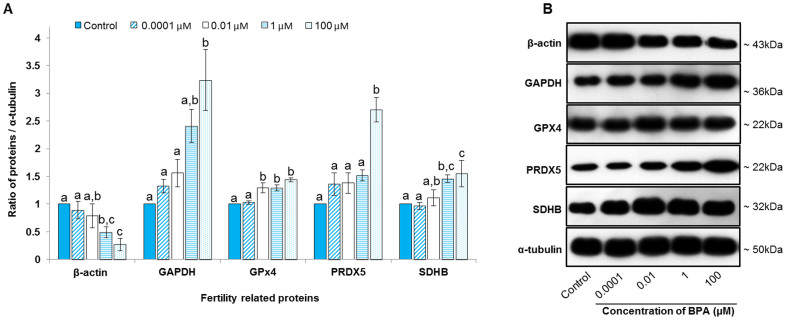
Effect of bisphenol-A (BPA) on selected fertility-related proteins in spermatozoa. (A) Density of β-actin, glyceraldehyde-3-phosphate dehydrogenase (GAPDH), glutathione peroxidase-4 (GPX4), peroxiredoxin-5 (PRDX5) and succinate dehydrogenase (SDHB) in the control (blue bar) and BPA-treated (stripped bar: 0.0001 μM; open bar: 0.01 μM; straight stripped bar: 1 μM; and dotted bar: 100 μM) spermatozoa. (B) Selected fertility-related proteins were probed with specific antibodies and detected: β-actin, approximately 43 kDa; GAPDH, approximately 36 kDa; GPX4, approximately 22 kDa; PRDX5, approximately 22 kDa; and SDHB, approximately 32 kDa. Lane 1: control; lane 2: 0.0001 μM BPA; lane 3: 0.01 μM BPA; lane 4: 1 μM BPA; and lane 5: 100 μM BPA. Data are presented as mean ± SEM (n = 3). Values with different superscript characters (^a,b,c^) indicate significant difference between the control and treatment groups as determined by one-way ANOVA (*P* < 0.05).

In this study, we chose several important fertility-related proteins (cytoskeletal β-actin, the glycolytic enzyme GAPDH, the electron transport chain enzyme SDHB, and stress response proteins GPX4 and PRDX5) to examine their functions in spermatozoa by western blotting due to BPA exposure. β-actin was effectively down-regulated by BPA, whereas the abundance of other proteins increased dose-dependently (Fig. 5A, B). Interestingly, significant changes in protein density occurred primarily after treatment with 100 μM BPA (Fig. 5A, B).

### Functional analysis of fertility-related proteins

**Figure 6:**
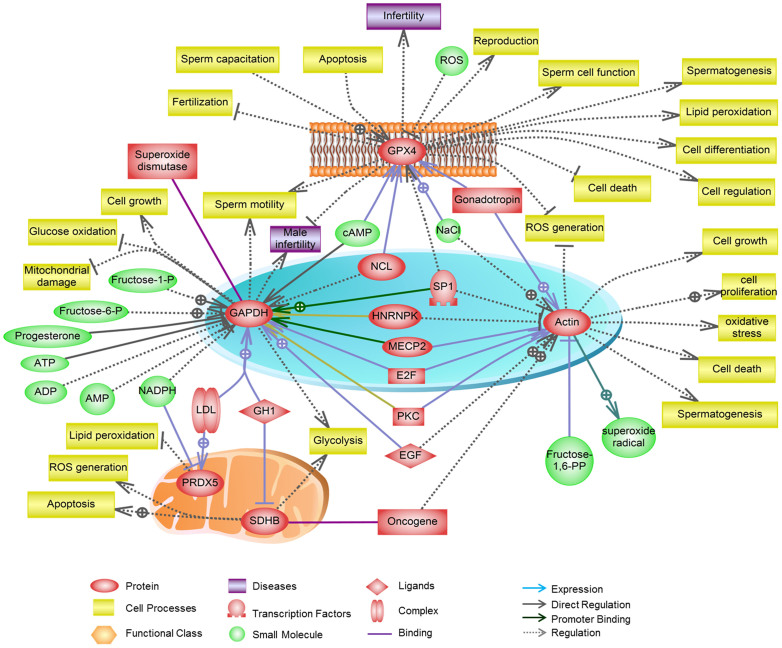
Pathways regulated by selected fertility-related proteins in spermatozoa as predicted by Pathway Studio software. Regulated processes are represented by backgrounds of different colors (indicated in the figure). Regulatory events are displayed using arrows and documented by the figure, and were supported by the literature. The references linked to each protein were generated by the software and are not listed.

An additional objective of the present study was to evaluate the functional interactions of the selected fertility-related proteins in spermatozoa. Detailed analysis of the cellular processes influenced by the selected proteins was performed using Pathway Studio version 9.0 (Fig. 6). The selected fertility-related proteins were putatively involved to areas related to sperm function, including ROS and energy metabolism, motility, hyperactivation, and the acrosome reaction, as well as cell growth, spermatogenesis, male fertility, and pathological conditions.

## Discussion

Estrogenic EDs like BPA are capable of binding with receptors on spermatozoa and thus affect sperm functions^11^,^12^. We used a comprehensive *in vitro* testing system to determine whether BPA interferes with sperm function, fertilization, and embryo development. Additionally, to evaluate the manner in which multiple proteins function in spermatozoa under BPA exposure, we monitored the functions of selected fertility-related proteins by western blotting. Furthermore, we explored the functional interactions of these proteins in spermatozoa in the context of male fertility and other health conditions.

Our results demonstrated that 100 μM BPA significantly decreased sperm motility and motion kinematic parameters following 6 h of incubation (Fig. 1A, B). Similar effects of BPA on sperm motility have been shown in rat^4^ and mice^9^ *in vivo*; however, *in vitro* exposure data on mammalian species have not been reported. Accordingly, decreased motility after exposure to 100 μM BPA was associated with decreased ATP levels in spermatozoa (Fig. 1C). The energy (ATP) used for sperm motility is mostly contributed by mitochondrial respiration^21^; therefore, if mitochondrial respiration does not produce sufficient ATP, sperm motility might decrease.

Capacitation and the acrosome reaction are prerequisites for oocyte fertilization by spermatozoa^17^,^26^, and both events are linked with modifications in plasma membrane fluidity, intracellular ionic concentrations, cAMP, tyrosine phosphorylation, and PKA activity^29^. Here, we have shown that the addition of BPA to spermatozoa leads to a dose-dependent induction of the acrosome reaction, with a particularly marked effect produced by 100 μM BPA (Fig. 2B). It has been reported that exposure of human spermatozoa to 1 μM of BPA for 2 h did not affect the acrosome reaction^30^; this difference with our results was likely due to differences in species, BPA concentrations, and the time of incubation. Additionally, we demonstrated that increased PKA and tyrosine phosphorylation activity accompany the acrosome reaction in spermatozoa (Fig. 3A, C). Indeed, PKA activity directly affects the tyrosine phosphorylation status of sperm proteins during capacitation and the acrosome reaction^25^,^29^,^31^. Therefore, taken together with previously published results, our findings suggest that high concentrations of BPA modulate cellular tyrosine phosphorylation by regulating PKA activity, which mediates the acrosome reaction.

Another novel finding of this study is the significant inhibitory effect of 100 μM BPA on fertilization and early embryonic development (Fig. 4B), which might be mediated by decreased sperm motility or premature triggering of the acrosome reaction. It has been reported that spermatozoa must exhibit proper movement in fluids to reach and fertilize the egg; thus, motility is a prime factor in successful conception^13^. However, the acrosome reaction is also important for fertilization^26^. Other studies have suggested that a premature acrosome reaction changes mitochondrial function, motility, and chromatin decondensation, and ultimately affect sperm viability and fertility^32^,^33^. A similar conclusion was made by Oliveira et al.^34^ and Rahman et al.^25^ after exposure of spermatozoa to the lead chloride and sodium nitroprusside, respectively. Collectively, our findings support the notion that a high concentration of approximately 100 μM BPA can produce a profound effect on critical sperm functions and fertilization, which has considerable implications for male fertility.

Recently, proteomic approaches have identified several proteins in spermatozoa^15^,^35^. However, individual protein functions need to be assessed to allow for better understanding of the complex mechanisms involved in sperm function^16^. In this study, we investigated a group of selected fertility-related proteins in spermatozoa under BPA exposure, and found that β-actin was down-regulated in spermatozoa by high concentrations of BPA, whereas other proteins, including GAPDH, GPX4, PRDX5, and SDHB, were up-regulated (Fig. 5A, B). Protein down-regulation in spermatozoa due to environmental changes *in vitro* is well established^36^,^37^; however, the manner in which protein levels are increased due to environmental changes remains to be elucidated.

Mature spermatozoa are thought to have lost their functional ability in transcription and translation, and are therefore incapable of protein synthesis^38^,^39^. However, some reports suggest that spermatozoa are capable of using mRNA transcripts for protein translation during their functional maturation^40^,^41^. This discrepancy aside, it is well accepted that spermatozoa acquire their functionality via post-translational protein modifications such as phosphorylation^37^,^38^,^39^,^40^,^41^,^42^. Protein phosphorylation is a common cellular mechanism whereby the addition of a negatively charged phosphate can alter the conformation of a target protein, and thus regulate its function^43^. Although serine, threonine, and tyrosine protein phosphorylation in spermatozoa have been studied, tyrosine phosphorylation has been shown to be a crucial, key activator of spermatozoa signaling pathways^44^. In this study, BPA increased tyrosine phosphorylation (Fig. 3C), which could mediate changes in the abundance of particular phosphorylated proteins. Taken together with a recent study conducted by Wang et al.^37^, our results suggest that exposure to environmental toxicants like BPA induces protein down-regulation and phosphorylation in spermatozoa, ultimately inhibiting their ability to function.

Actin is one of the most abundant cytoskeletal proteins in spermatozoa, and it is localized in the acrosome, equatorial region, and tail^18^. The localization of actin in the tail indicates its function in sperm motility, whereas actin in the head may be involved in the acrosome reaction^18^. Polymerization of globular actin to filamentous actin is critical for capacitation, and this process is regulated by PKA activity and protein tyrosine phosphorylation^18^. Filamentous actin should undergo depolymerization early in the acrosome reaction. In the present study, the density of β-actin was decreased in spermatozoa due to BPA exposure (Fig. 5A, B), suggesting that BPA down-regulated β-actin in spermatozoa, and thus affected sperm motility, the acrosome reaction, and fertility. It has been reported that decreased actin abundance is associated with metastatic melanoma in mice^45^ and prostatic carcinoma in rats^46^.

GPX4 and PRDX5 are antioxidant enzymes that are predominantly expressed in spermatozoa as a response to stress. In somatic cells, GPX1, GPX4, PRDX3, and PRDX5 are responsible for consumption of approximately 99.9% of mitochondrial H_2_O_2_^47^. In mammalian spermatozoa, mitochondria are the principle site of ROS generation, which is strongly associated with male infertility^48^. Thus, failure of the antioxidant defense system in mitochondria to remove H_2_O_2_ leads ROS concentrations to reach toxic levels, and ultimately compromises normal sperm function and fertility. Therefore, the elevated levels of GPX4 and PRDX5 observed in the present study (Fig. 5A, B) were assumed to offer the sperm cells a survival advantages (ROS levels remained unchanged, see Supplemental Material, Fig. S2) in a BPA-containing microenvironment. Consistent with our findings, overexpression of PRDX5 has been reported in breast carcinoma^49^, hepatocellular carcinoma^50^, and mesothelioma cells^51^.

GAPDH and SDHB are involved in glycolysis and the electron transport chain, respectively, and regulate sperm motility and male fertility^21^,^22^. Disruption of GAPDH expression by gene targeting can be used to block glycolysis in spermatozoa, which results in severe motility defects^21^. Xue et al.^22^ reported that the motility and viability of sperm were positively correlated with mitochondrial SDHB. Therefore, GAPDH and SDHB could influence mitochondrial function in spermatozoa and may serve as markers of sperm quality and male fertility. In the present study, BPA dose-dependently increased GAPDH and SDHB abundance; significant changes in protein abundance were produced by exposure to higher concentrations of BPA (Fig. 5A, B). These results suggest that high concentrations of BPA alter the function of GAPDH and SDHB, and could thus affect male fertility. Our results do not provide a clear explanation as to the manner in which ATP/motility are reduced by increased levels of GAPDHS/SDHB caused by exposure to 100 μM BPA. However, the increased GAPDH/SDHB may have been insufficient to overcome BPA-mediated toxicity, and/or the substantial increase in expression resulted in atypical enzyme functioning. Enhanced glycolysis has been well established in cancer cells^52^. Because BPA may be carcinogenic^3^, there might be a relationship between BPA-mediated increases in the levels of glycolytic enzymes and the occurrence of cancers in somatic cells. SDHB dysfunction is strongly associated with somatic cell tumorigenesis^53^. However, further studies are necessary to resolve this issue.

## Conclusions

To our knowledge, this is the first comprehensive description of the *in vitro* effects of BPA on mouse spermatozoa. BPA at concentrations of 0.0001, 0.01, and 1 μM did not produce significant or partial toxic effects on spermatozoa; however, 100 μM BPA affected motility parameters, the acrosome reaction, fertilization, and early embryonic development, which are closely associated with down-regulation and phosphorylation of fertility-related proteins in spermatozoa. BPA-induced changes in the abundance of fertility-related proteins may be associated with increased health risk (Fig. 6).

## Methods

### Media and chemicals

All chemicals and reagents were purchased from Sigma-Aldrich (St. Louis, MO, USA), unless otherwise specified. Modified Tyrode's medium was used as a basic medium (BM), and prepared as previously described^25^,^54^. The culture medium was pre-incubated 1 day prior to the experiment and supplemented with bovine serum albumin (BSA; 4 mg/mL) to induce capacitation. The BPA stock solution was diluted with dimethyl sulfoxide (DMSO) and stored in conical tubes at room temperature (RT). The stock solution was diluted with BM to achieve desired final concentrations of 0.0001, 0.01, 1, and 100 μM. This concentration range was chosen because high doses of hormones (and estrogen mimics like BPA) can act through negative feedback loops to inhibit their own effects, and low doses are sufficient to exert their primary effects^55^. Additionally, exposure to environmentally relevant doses of BPA (approximately 1–3 nM) advances early and postnatal embryonic development^56^, whereas high concentrations (100 μM) significantly decreased the development rate^57^.

### Sperm collection, preparation, and exposure to BPA

All animal procedures were conducted according to the guidelines and were approved by the Institutional Animal Care and Use Committee of Chung-Ang University, Seoul, Korea. Experimental mice were kept in a room with controlled temperature (22 ± 2°C), ventilation, and lighting (12-h light/dark cycles) and were provided *ad libitum* access to laboratory feed (Cargill Agripurina, Seongnam, Korea) and water. Spermatozoa were collected from sexually mature male ICR mice (Nara Biotech, Seoul, Korea) using a standard procedure^25^,^31^. Briefly, both cauda epididymides from each mouse were collected and the associated fat was removed. The sample was then placed on a piece of filter paper to remove any liquid. The cauda epididymides were placed in cell culture dishes with BM, and punctured using a sterile needle to facilitate the release of spermatozoa. After preincubation, the sperm suspension was incubated in BM (containing 4 mg/mL BSA) supplemented with 0.0001, 0.01, 1, and 100 μM of BPA for 6 h in air at 37°C. The control group consisted of DMSO-treated spermatozoa that did not contain BPA. For each of the independent experiments, three 3 male mice per replicate were considered.

### Assessment of sperm motility and motion kinematic parameters

Sperm motility and kinematic parameters were measured using computer-assisted sperm analysis (CASA) (SAIS-PLUS version 10.1; Medical Supply, Seoul, Korea) as previously described^29^. The 10× phase-contrast objective was used by the SAIS software to relay and analyze the spermatozoa, and 5 fields from each sample were randomly selected for evaluation of the movement of at least 250 sperm.

### Measurement of cellular reactive oxygen species (ROS)

Cellular ROS content was measured using the oxidation-sensitive fluorescent dye 2′,7′-dichlorofluorescin diacetate (DCFDA; Abcam, Cambridge, England) as previously described^25^. Briefly, after 6 h of incubation, the samples were washed and resuspended in 1 mL DCFDA mix and incubated at 37°C for 30 min. Samples were resuspended in supplemental buffer after a second wash. Finally, the cell suspension (500 μL) was seeded in a dark 96-well plate. Fluorescence was detected with a microplate fluorometer (Gemini Em; Molecular Devices, Sunnyvale, CA, USA) and quantified with SoftMax Pro 5 (Molecular Devices). The fluorescence of all treatment groups and the control group was calculated as a ratio (485 nm/535 nm) and reported as the ratio of the fluorescence of the BPA-treated samples to that of the control samples.

### Measurements of intracellular ATP generation

Cellular ATP was quantitatively detected using an ATP Bioluminescence Assay Kit (CLS II; Roche Molecular Biochemicals, Mannheim, Germany) according to the manufacturer's protocol and previously described methods^25^,^31^. Briefly, the treated cells were diluted (10^5^–10^8^ cells/μL), and 25 μL of the diluted cells was added to each well of a 96-well plate, to which 25 μL of cell lysis reagent was added. The plate was incubated at RT for 5 min, diluted ATP (50 μL) and luciferase reagent (50 μL) were added, after which luminescence was detected using a Microplate Multimode Reader (GloMax®-Multi Microplate Multimode Reader; Promega, Madison, WI, USA).

### Assessment of sperm capacitation status

The capacitation status of the spermatozoa following BPA treatment was determined by a dual-staining method (combined Hoechst 33258/chlortetracycline fluorescence assessment) as described previously^29^,^31^. The spermatozoa were observed under a Microphot-FXA microscope (Nikon, Tokyo, Japan) with epifluorescence illumination using ultraviolet BP 340–380/LP 425 and BP 450–490/LP excitation/emission filters for H33258 and CTC, respectively. This dual-staining method analysis revealed 4 separate patterns among the spermatozoa, which were identified according to previously reported criteria^58^: dead (D pattern, blue fluorescence distributed uniformly over the sperm head), live non-capacitated (F pattern, bright green fluorescence distributed uniformly over the sperm head), live capacitated (B pattern, bright green fluorescence over the acrosomal region and a dark post-acrosomal region), and live acrosome-reacted (AR pattern, no fluorescence over the head, or green fluorescence only in the post-acrosomal region). At least 400 spermatozoa were evaluated per slide for each condition.

### In vitro fertilization

Eight- to 12-week-old B6D2F1/CrljOri hybrid female mice (Nara Biotech, Seoul, Korea) were super-ovulated by pregnant mare serum gonadotropin and human chorionic gonadotropin^29^,^31^. The oocytes were fertilized *in vitro* with control and BPA-treated spermatozoa according to previously described methods that were optimized for mice^25^,^29^. The fertilization rate was assessed by calculating the number of 2-cell embryos after 18 h of insemination. All embryos that developed to the blastocyst stage were counted.

### Western blot analysis of phospho-PKA substrates, tyrosine phosphorylation, and a selected group of 5 fertility-related proteins

Mammalian spermatozoa must undergo capacitation and the acrosome reaction to achieve successful fertilization^25^,^26^, and both of these events are associated with the activation of cAMP/PKA-dependent signaling, which is followed by up-regulation of protein tyrosine phosphorylation^25^,^27^. Therefore, the PKA substrates and tyrosine phosphorylation of sperm proteins were assayed in current study according to previously described methods^25^,^29^. Briefly, a sample containing 500 × 10^6^ cells/mL from the treated spermatozoa was electrophoresed on a 10% SDS polyacrylamide gel and transferred to a polyvinylidene fluoride membrane (GE Healthcare, Pittsburgh, PA, USA). The membranes were blocked in 3% ECL blocking agent (GE Healthcare). To detect PKA substrates, the membranes were incubated with an anti-phospho-PKA substrate rabbit polyclonal antibody (1:10,000; Cell Signaling Technology, Danvers MA, USA). The membranes were incubated with horseradish peroxidase (HRP)-conjugated goat anti-rabbit IgG (1:5,000; Abcam, Cambridge, England), and tyrosine phosphorylation was detected using an HRP-conjugated mouse monoclonal anti-phosphotyrosine antibody (PY20, 1:2,500, Abcam). Western blots were also used to detect the fertility-related proteins, such as β-actin, GAPDH, GPX4, PRDX5, and SDHB to evaluate their role in spermatozoa after BPA exposure. Because, intensive studies on individual proteins are necessary to investigate the molecular basis of sperm function due to BPA exposure. For the detection of protein levels following BPA treatment, the membrane was blocked with 5% skim milk blocking agent. The proteins from treated spermatozoa were immunodetected after an overnight RT incubation with an anti-β-actin mouse monoclonal antibody (HRP-conjugated), an anti-GAPDH mouse monoclonal antibody, an anti-GPX4 rabbit polyclonal antibody, an anti-PRDX5 rabbit polyclonal antibody, or an anti-SDHB rabbit polyclonal antibody (all from Abcam), each of which was diluted with blocking solution (1 μg/mL). After primary incubation, the membranes were incubated in blocking solution with HRP-conjugated goat anti-rabbit IgG (1:5,000; for GPX4, PRDX5, and SDHB; Abcam) or mouse IgG (for GAPDH) as a secondary antibody. α-Tubulin expression was determined using a monoclonal anti α-tubulin mouse antibody and used as an internal control for each protein. The proteins on the membranes were detected using enhanced chemiluminescence (ECL) reagents. All protein bands were scanned with a GS-800 calibrated imaging densitometer (Bio-Rad, Hercules, CA, USA) and analyzed with Quantity One software (Bio-Rad). Finally, the signal intensity ratios of the bands were calculated for phospho-PKA substrates, tyrosine phosphorylation species, β-actin, GAPDH, GPX4, PRDX5, and SDHB with respect to α-tubulin.

### Signaling pathway analysis

Pathway Studio (version 9.0, Ariadne Genomics, MD, USA) was used to predict the biological functions and signaling pathways associated with fertility-related proteins in spermatozoa^14^,^26^. The fertility-related proteins were entered into Pathway Studio in order to determine their associated signaling pathways, cellular functions, cellular regulation, and interactions with other proteins in spermatozoa.

### Statistical analysis

Data were analyzed using one-way ANOVA in SPSS (version 12.0; IBM Corp., Armonk, NY, USA) and Tukey's test was used to determine the significance of differences between each group. Student's two-tailed t-test was employed to compare changes in motility (time-dependent) between the control and treatment groups. P-values < 0.05 indicated statistical significance, and data are expressed as mean ± SEM.

## Author Contributions

M.S.R., W.S.K., J.S.L. and S.J.Y. performed the experiments. M.S.R. and W.S.K. analyzed the data and performed the artwork. M.S.R. and M.G.P. draft the manuscript. Both M.G.P. and B.Y.R. supervised the design of study and revised the manuscript. All authors critically reviewed the manuscript for intellectual content and gave final approval for the version to be published.

## Supplementary Material

Supplementary Information Supplementary Information
