# Supplementary material for: Bisphenol-A Affects Male Fertility via Fertility-related Proteins in Spermatozoa
Source: Sci Rep. 2015 Mar 16;5:9169. doi: 10.1038/srep09169 (PMC4360475; doi:10.1038/srep09169)
Supplement: Supplementary Information [file srep09169-s1.pdf]

Supplemental Material for: Rahman *et al*.

(Title)

Bisphenol-A Affects Male Fertility via Fertility-related Proteins in Spermatozoa

Md Saidur Rahman, Woo-Sung Kwon, June-Sub Lee, Sung-Jae Yoon, Buom-Yong Ryu, and Myung-Geol Pang

Department of Animal Science and Technology, Chung-Ang University, Anseong,

Gyeonggi-do 456-756, Republic of Korea

Running title: Bisphenol-A Suppresses Male Fertility *In Vitro*

Supplementary Figure Legends:

Figure S1. Differences in sperm motility with 100 µM of BPA compare to control in different time of exposure. Data are presented as mean ± SEM (4 replicates). Student's two-tailed *t*-test and one-way ANOVA were performed to determine the significance of differences between and within groups, respectively. Values with asterisks (*) indicate significant difference between the control and treatment groups.

Figure S2. Effect of bisphenol-A (BPA) on intracellular ROS levels in spermatozoa. Differences in ROS levels between the control and BPA-treated samples. Data are presented as mean ± SEM (4 replicates).

Supplementary Figures

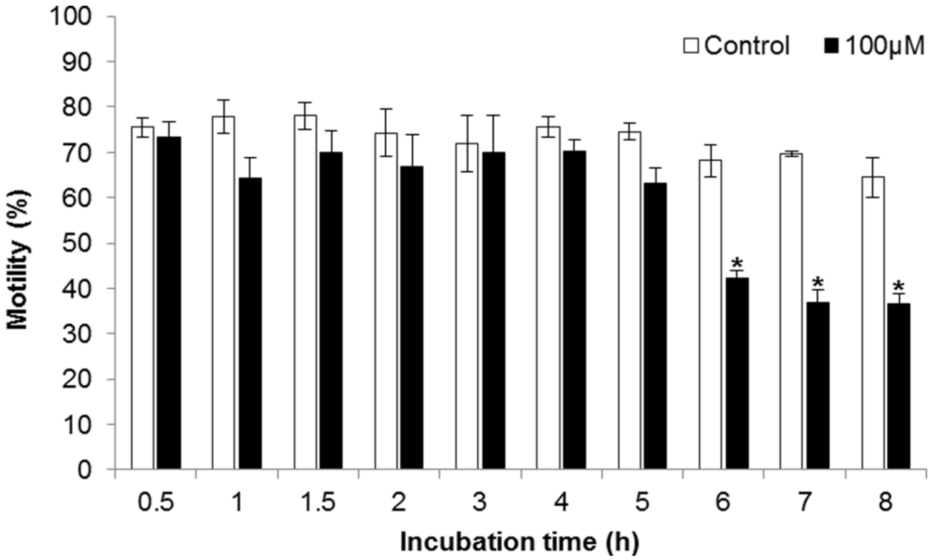

Supplementary Figure-S1

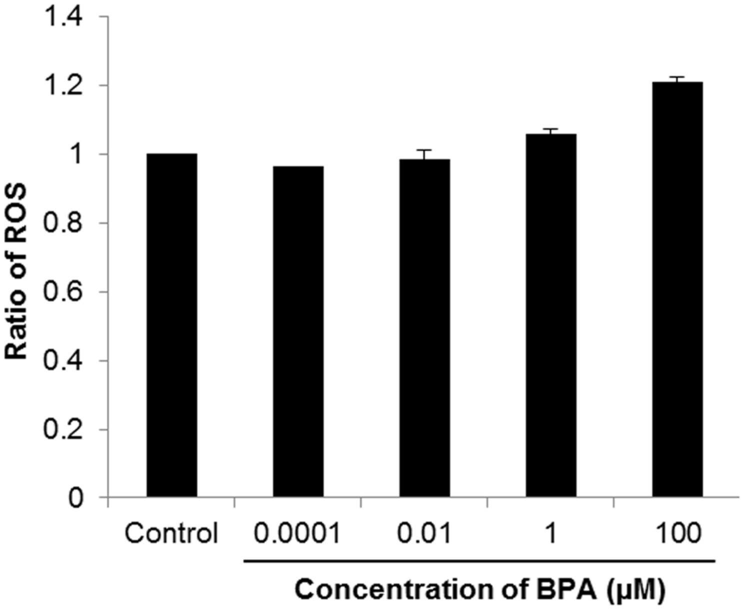

Supplementary Figure-S2
